# Les dermatoses génitales: profil épidémiologique et Clinique

**DOI:** 10.11604/pamj.2014.18.240.4797

**Published:** 2014-07-23

**Authors:** Siham Lakjiri, Mariame Meziane, Sara Elloudi, Ousmane Sy, Chakib Nejjari, Fatima Zahra Mernissi

**Affiliations:** 1 Service de Dermatologie, CHU Hassan II, Fès, Maroc

**Keywords:** Dermatoses génitales, épidémiologie, organes génitaux, genital dermatoses, epidemiology, genitals

## Abstract

La pathologie cutanéo-muqueuse des organes génitaux externes (OGE) est très variée. Le but de cette étude est de décrire le profil épidémiologique de cette dermatose au CHU Hassan II de FES. Il s'agit d'une étude prospective descriptive menée au sein du service de dermatologie de CHU Hassan II de FES s’étalant sur une période de 22 mois. Un total de 350 patients ont été inclus dans cette étude, 179 femmes (51%) et 171 hommes (49%). L’âge moyen de nos patients était de 39 ans, 69% étaient d'un niveau socio- économique faible. La plupart des patients étaient mariés (63,4%). Les étiologies retrouvées étaient dominées par l’étiologie virale (92 cas: 26%), suivi par le vitiligo (13%), l'origine fongique en 3ème classe avec un pourcentage de 11%, suivie par le psoriasis (8%), et d'autres étiologies diverses: Lichen plan et le lichen scléreux (7,7%), l'eczéma (5%), les tumeurs malignes (5%), dermatoses bulleuses, les maladies bactériennes et parasitaires. Avec 350 cas diagnostiqués en vingt-deux mois, notre étude montre que les dermatoses des OGE ne sont pas rares dans notre service. L'analyse des résultats de notre série montrait la survenue de dermatoses génitales chez toutes les tranches d’âge y compris les nourrissons avec une prédominance chez la population sexuellement active autour de 30 à 40 ans. Les dermatoses génitales d'origine virale étaient les plus retrouvés dans notre étude et cela était prédominant dans la population adulte la plus sexuellement active, cela peut être expliqué par le niveau socio-économique bas, la promiscuité et la multiplicité des partenaires sexuels. La plupart des causes ont un pronostic favorable, mais elles ont un impact psychologique important. Cette étude souligne l'importance de diagnostiquer les dermatoses génitales et réfute l'idée fausse générale que toutes les démangeaisons génitales sont le résultat d'une infection fongique notamment candidosique.

## Introduction

La dermatologie génitale étudie les dermatoses cutanéomuqueuses des organes génitaux externes (OGE). De nombreuses affections peuvent toucher les OGE [1, 2] qu'il s'agisse d'une affection de cause externe, qui, lorsqu'elle est infectieuse, peut être ou non sexuellement transmissible, ou bien d'un processus tumoral, ou encore de la localisation génitale d'une dermatose ou d'une affection générale. Ces dermatoses sont abordées avec beaucoup de pudeur dans nos sociétés Arabes. L'objectif de cette étude était de décrire les profils épidémiologique et clinique des dermatoses des organes génitaux externes au service de dermatologie de CHU Hassan II de FES.

## Méthodes

Il s'agit d'une étude prospective descriptive qui a permis de colliger 350 cas de dermatoses génitales recrutés au service de dermatologie couvrant une période de 22 mois de Janvier 2012 à Octobre 2013. Les critères d'inclusion étaient tout patient se présentant en consultation ou en hospitalisation de dermatologie pour une symptomatologie génitale présentant des lésions au niveau des OGE. Les critères d'exclusion étaient les MST (ulcérations génitales et les urétrites).

Pour les patients inclus dans l’étude ont été recueillis sur une fiche d'exploitation préétablie. Les paramètres suivants ont été étudiés: l’âge, le sexe, l’état patrimonial, les antécédents personnels médicaux et sexuels et les antécédents familiaux, le délai de consultation (la durée d’évolution avant la consultation), le siège des lésions, leur nombre et leur taille, l'examen du reste des téguments, examen dermoscopique, examen à la lumière de wood, les sérologies HIV, sérologie TPHA/VDRL. La biopsie et l'examen histopathologique de l’échantillon ont été effectués lorsqu'ils étaient jugés nécessaires pour confirmer le diagnostic. Ont aussi été recueillis les différents traitements administrés (en fonction du diagnostic) et l’évolution. Les données ont été saisies sur le logiciel Excel et analysées sur le logiciel SPSS Statistics version 20.

## Résultats

Un total de 350 malades atteints d'une dermatose génitale a été inclus dans cette étude. Il s'agissait de 179 femmes soit 51% des cas et 171 hommes. L’âge des patients variait d'un an à 103 ans, avec un âge moyen de 39 ans. 69% des cas avaient un bas niveau socio-économique. Deux cents vingt-deux (63,4%) d'entre eux étaient mariés. Le symptôme le plus retrouvé était le prurit dans 51% des cas, d'autres plaintes ont été retrouvées notamment la douleur, la dyspareunie et le suintement, certains patients ont eu plus d'une plainte. La localisation vulvaire était le site le plus retrouvé chez les femmes soit 61,45%, chez les hommes: le scrotum était atteint dans 53,5% des cas suivis par la verge dans 50,3% des cas, le pubis était atteint dans 25,4% chez les deux sexes. La localisation anale associée était retrouvée chez 14% des malades.

Sur le plan sérologique, 5 patients avaient une sérologie HIV positive et 10 patients avaient une sérologie syphilitique positive. L'indication de la biopsie a été posée chez 17% des malades.

**Figure 1:**
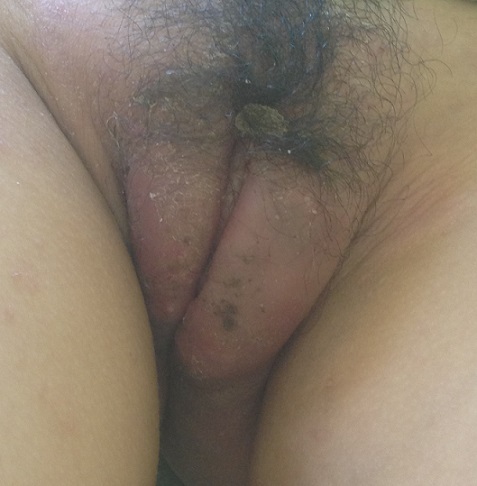
Hypertrophie vulvaire de maladie de Crhon

**Figure 2:**
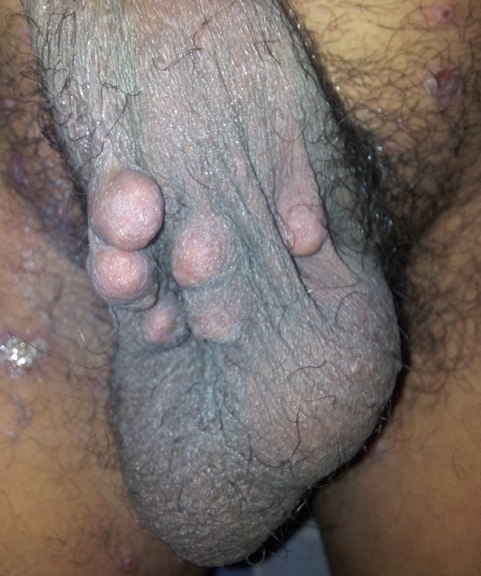
Nodules scrotaux

**Figure 3:**
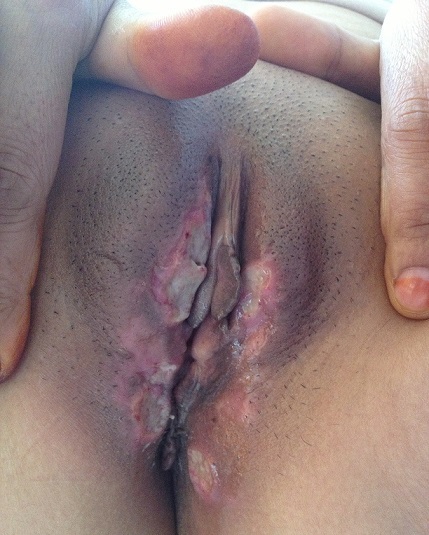
Aphtes génitaux de la maladie de Behcet

**Figure 4:**
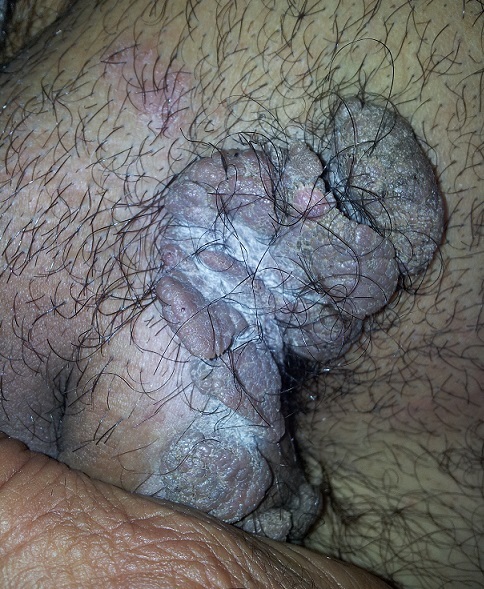
Dermatophytie avec un surinfection bactérienne chez une patiente sous chimiothérapie

**Figure 5:**
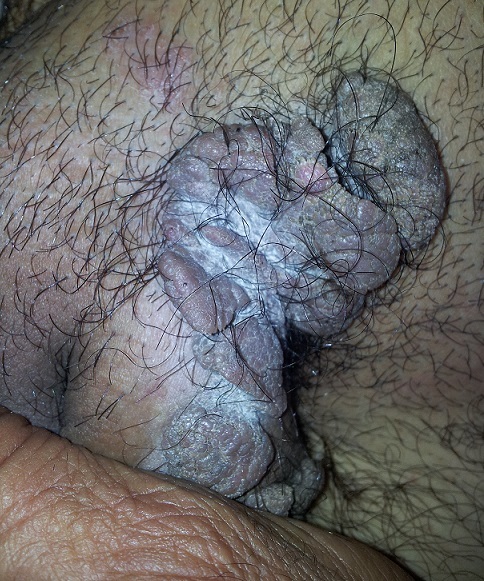
Condylomes génitaux

**Figure 6:**
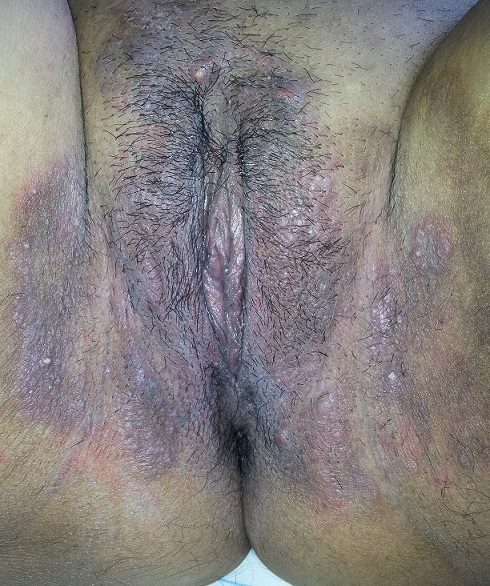
Vitiligo génital

Un total de 16 types de dermatoses génitales différentes ont été observées dans notre étude qui étaient résumées dans leTableau 1. elles se sont prédominées par l’étiologie virale (Figure 1) dont les condylomes étaient la pathologie virale la plus retrouvée, suivie par le vitiligo (Figure 2), l'origine mycosique occupe la 3ème place (Figure 3), puis les autres étiologies variées: psoriasis, lichen plan et lichen scléreux, eczéma, pathologie tumorale (Figure 4), les dermatoses bulleuses auto-immunes (DBAI), les affections bactériennes et parasitaires, les maladies de système (Figure 5) puis d'autres pathologies variées (Figure 6) Les traitements ont été instaurés en fonction de l’étiologie avec une amélioration dans 67% des cas et 16% au début du traitement.

**Tableau 1:** Types de dermatoses génitales observées dans notre étude

Pathologies	Femmes (179)	Hommes (171)	Filles (27)	Garçons (24)	Total
	n (%)	n (%)	n (%)	n(%)	n (%)
Infectieuses	73 (40.78)	79 (46.19)	7 (25.9)	10 (42)	152 (43.4)
Virale	41 (22.9)	51 (29.89)	5 (18.5)	3 (12.5)	92 (26)
Mycosique	22(12.30)	15 (8.8)	2 (7.4)	3 (12.5)	37 (11)
Parasitaire	1(0.5)	10 (5.8)	-	4 (16.66)	11 (3.14)
Bactérienne	9 (5)	3 (1.75)	-	-	12 (3.43)
Inflammatoire	39 (21.78)	36 (21)	11 (41)	3 (12.5)	75 (21)
Psoriaisis	10 (5.6)	20 (11.7)	4 (14.8)	3 (12.5)	30 (8)
Lichen plan	8 (4)	7 (4)	1 (3.7)	-	16 (4.6)
Lichen scléreux	10 (5.6)	-	4 (14.8)	-	14 (4)
Eczéma	9 (5)	9 (5.3)	2 (7.4)	-	18 (5)
Auto-immunes	35 (19.55)	30 (17.5)	6 (22.22)	2 (8.33)	64 (18.3)
Vitiligo	23 (12.8)	24 (14)	6 (22.22)	2 (8.33)	47 (13)
Maladies de système	3 (2)	3 (1.75)	-	-	6 (1.7)
DBAI	9 (5)	3 (1.75)	-	-	11 (5)
Pathologie tumorale	12 (6.7)	9 (5.26)	2 (7.4)	2 (8.33)	17 (5)
Autres	22 (12.30)	19 (11)	1 (3.7)	7 (29)	46 (13)

DBAI: dermatoses bulleuses auto-immunes

## Discussion

Il n'existe pas d’études complètes sur le profil des dermatoses non vénériennes d'un pays en développement comme le nôtre d'où l'intérêt de notre étude. Avec 350 cas diagnostiqués en vingt-deux mois, notre étude montre que les dermatoses des OGE ne sont pas rares dans notre population. Les dermatoses des OGE sont des affections cutanées à localisation génitale, elles comprennent un éventail de maladies dont l’étiologie est variée [1, 2]. Ces dermatoses peuvent être associées à un grave traumatisme psychologique et une peur dans l'esprit des patients. Par conséquent, il est d'une immense importance de connaitre ces dermatoses génitales et les diagnostiquer tôt pour soulager le patient. Les dermatoses génitales ne sont pas toujours transmises sexuellement. Ceux qui ne sont pas sexuellement transmissibles sont considérés comme des dermatoses non vénériennes des OGE [3].

L'analyse des résultats de notre série montrait la survenue de dermatoses génitales chez les 2 sexes avec une légère prédominance féminine. Par ailleurs, elles touchaient toutes les tranches d’âge y compris les nourrissons avec une prédominance chez la population la plus sexuellement active autour de 30 à 40 ans; des résultats similaires étaient retrouvés dans une étude réalisée en *Guinée* [4] à propos de 102 malades.

Les mariés étaient les plus exposés avec un pourcentage de 63.4% contrairement à la série *Guinéenne* [4], malgré l'exclusion des MST dans notre série, et ceci peut être expliqué par la diversité des étiologies.

Le retard diagnostic avec un délai de consultation allant jusqu’à 36 mois peut être expliqué par le bas niveau socioculturel de la plupart des patients (69%) qui ne consultent pas devant la gêne essentiellement modérée de certaines pathologies avec recours fréquent à l'automédication et aux traitements traditionnels et à la honte de certains d'autres.

Les dermatoses génitales ne sont pas toujours de type IST, elles comprennent un éventail de maladies dont l’étiologie est variée: inflammatoire (psoriasis, dermatite séborrhéique, lichen..), auto-immune (vitiligo), maladies de système (behçet, MICI...) [3, 5].

Les dermatoses génitales d'origine virale étaient les plus retrouvés dans notre étude malgré l'exclusion des MST et cela était prédominant dans la population adulte la plus sexuellement active, cela peut être expliqué par le niveau socio-économique bas, la promiscuité et la multiplicité des partenaires sexuels. Cependant d'autres pathologies peuvent être retrouvées notamment inflammatoires, immunitaires’

Il ne faut pas oublier que le diagnostic des lésions cancéreuses des organes génitaux externes en particulier et les autres dermatoses en général de l'homme est encore beaucoup trop tardif en raison des sentiments de honte et de pudeur vécus par les patients, mais aussi en raison de l'absence d'examen systématique de cette zone anatomique par les médecins [1]. La localisation des dermatoses génitales chez les femmes prédominent au niveau des grandes et petites lèvres dans notre série ce qui rejoint l’étude indienne à propos de 120 patients avec un pourcentage de 92% au niveau des grandes lèvres et 48% au niveau des petites lèvres [2]. Les dermatoses génitales retrouvées chez les femmes dans notre étude sont prédominées par les dermatoses d'origine virale suivie par le vitiligo puis l'origine mycosique, cela n'est pas le cas dans la littérature où elles sont prédominées par le lichen scléreux suivi par le vitiligo puis l'origine mycosique et les autres étiologies [2, 6, 7].

L'atteinte génitale masculine de lichen scléreux est assez fréquente puisque 15% des dermatoses péniennes de deux séries [8, 9] sont des Lichen scléreux et tous sont des malades non circoncis, contrairement à notre étude où on n'a trouvé aucun cas de lichen scléreux génital et cela est probablement expliqué par la circoncision de notre population musulmane.

Les lésions anales associées surviennent chez 14% des cas, cela était conforme aux résultats de la littérature [2] d'où l'intérêt d'un examen systématique des autres muqueuses. La plupart des étiologies sont de pronostic favorable mais elles ont un retentissement psychologique important [1]. Le retentissement psychologique des dermatoses des OGE est très fréquent en raison de sentiments complexes dont l'atteinte de la virilité [1, 2], en particulier les femmes [10]. Il doit être pris en compte par une écoute attentive au-delà du simple interrogatoire médical.

## Conclusion

Les dermatoses génitales constituent un motif fréquent de consultation. Une bonne connaissance de ces dermatoses reste nécessaire pour une meilleure prise en charge des malades. Et elles méritent une attention particulière car pouvant être très invalidantes et susceptibles d'entrainer des troubles psychologiques graves et des conséquences non négligeables sur la sexualité. Cette étude souligne l'importance de diagnostiquer les dermatoses génitales et réfute l'idée fausse générale que toutes les démangeaisons génitales sont le résultat d'une infection fongique notamment candidosique. D'où la nécessité de formations des médecins généralistes sur les dermatoses génitales et cibler la population jeune.

## References

[1] Chaine B, Janier M (2005). Dermatoses génitales masculines. EMC - Dermatologie-Cosmétologie.

[2] Singh N, Thappa DM, Jaisankar TJ, Habeebullah S (2008). Pattern of non-venereal dermatoses of female external genitalia in South India. Dermatol Online J..

[3] Khaitan BK, Sharma VK (2003). Non-venreal diseases of genitalia. Sexually Transmitted Diseases and AIDS.

[4] Soumah MM, Keita M, Diane B-F, Tounkara TM, Balde H (2012). Les dermatoses des organes génitaux: profils épidémiologique et clinique au service de dermatologie-MST de l'hôpital national Donka, CHU de Conakry. Ann Dermatol Venereol..

[5] Meffert JJ, Davis BM, Grimwood RE (1995). Lichen sclerosus. J Am Acad Dermatol..

[6] Sullivan AK, Straghair GJ, Marwood RP, Staughton RC, Barton SE (1999). A multidisciplinary vulva clinic: the role of genitor-urinary medicine. J Eur Acad Dermatol Venereol..

[7] Cheung ST, Gach JE, Lewis FM (2006). A retrospective study of the referral patterns to a vulval clinic: highlighting educational needs in this subspecialty. J Obstet Gynaecol..

[8] Mallon E, Hawkins D, Dinneen M, Francics N, Fearfield L, Newson R, Bunker C (2000). Circumcision and genital dermatoses. Arch Dermatol..

[9] Hillman RJ, Walker MM, Harris JR, Taylor-Robinson D (1992). Penile dermatoses: a clinical and histopathological study. Genitourin Med..

[10] Meeuwis KA, de Hullu JA, van de Nieuwenhof HP (2011). Quality of life and sexual health in patients with genital psoriasis. Br J Dermatol..

